# Long-term results of in-the-bag primary intraocular lens implantation in children younger than 24 months

**DOI:** 10.5935/0004-2749.20210045

**Published:** 2021

**Authors:** Camila Ribeiro Koch, Newton Kara-Junior, Marcony Rodrigues Santhiago, Marta Morales

**Affiliations:** 1 Universidade de São Paulo, São Paulo, SP, Brazil; 2 Ophthalmology Unit, Sant Joan de Déu Hospital, Barcelona, Spain

**Keywords:** Pediatric cataract, Intraocular lens, Primary IOL implantation, Myopic shift, Congenital cataract, Catarata pediátrica, Lente intraocular, Implante primário LIO, Mudança miópica, Catarata congênita

## Abstract

**Purpose:**

The purpose of this study was to analyze the safety of primary intraocular lens implantation in a large number of eyes in children aged <24 months.

**Methods:**

The medical records of patients aged 5-24 months, who underwent primary intraocular lens implantation in the capsular bag, were reviewed. A foldable three-piece acrylic intraocular lens was implanted by the same surgeon using a single surgical technique. Patients who had <1 year of follow-up after the surgery were excluded. The main outcome measurements included visual acuity, myopic shift, follow-up complications, and additional surgeries.

**Results:**

Sixty-eight patients (93 eyes) were analyzed. The mean age of the patients at the time of surgery was 15.06 ± 6.19 months (range: 5-24 months), and the spherical equivalent 1 month after surgery was 3.62 ± 2.32 D. After 5.67 ± 3.10 years, the spherical equivalent was -0.09 ± 3.22 D, and the corrected distance visual acuity was 0.33 ± 0.33 and 0.64 ± 0.43 logMAR in bilateral and unilateral cases, respectively (p=0.000). The highest myopic shift was observed in infants who underwent surgery at ages 5 and 6 months. The most frequent complications included visual axis opacification and corectopia. Glaucoma and retinal detachment were not reported.

**Conclusion:**

Primary in-the-bag intraocular lens implantation in children aged 5-24 months is safe, and is associated with low rates of adverse events and additional surgery.

## Abstract (pt)

**Objetivo:**

O objetivo deste estudo foi analisar a segurança do implante de lente intraocular primária em um grande número de olhos em crianças <24 meses.

**Métodos:**

Foram revisados os prontuários de pacientes com idade entre 5-24 meses, submetidos a implante primário de lente intraocular no saco capsular. Uma lente intraocular acrílica de três peças dobrável foi implantada pelo mesmo cirurgião usando uma única técnica cirúrgica. Pacientes que tiveram <1 ano de acompanhamento após a cirurgia foram excluídos. Os principais resultados incluíram medidas de acuidade visual, mudança miópica, complicações pós operatórias e cirurgias adicionais.

**Resultados:**

Foram analisados 68 pacientes (93 olhos). A média de idade dos pacientes no momento da cirurgia foi de 15,06 ± 6,19 (5 a 24) meses, e o equivalente esférico 1 mês após a cirurgia foi de 3,62 ± 2,32 D. Após 5,67 ± 3,10 anos, o equivalente esférico foi de -0,09 ± 3,22 D, e a acuidade visual corrigida à distância foi de 0,33 ± 0,33 e 0,64 ± 0,43 logMAR em casos bilaterais e casos unilaterais, respectivamente (p=0,000). A maior mudança míopica foi observado em bebês submetidos à cirurgia aos 5 e 6 meses de idade. As complicações mais frequentes incluíram opacificação do eixo visual e corectopia. Glaucoma e descolamento de retina não foram relatados.

**Conclusão:**

O implante primário de lente intraocular no saco capsular em crianças de 5-24 meses é seguro e está associado à baixas taxas de eventos adversos e cirurgias adicional.

## INTRODUCTION

Primary intraocular lens (IOL) implantation can prevent additional surgery for secondary IOL implantation and provides partial optical correction at all times^(1-3)^. Safe outcomes have been demonstrated in patients aged >2 years; however, for patients younger than this age^(4-7)^, there is no consensus regarding the appropriate month of life to perform IOL implantation since more inflammation and adverse events are expected in infants^(8-11)^.

Although advances in IOL designs and surgical techniques have led to lower rates of visual axis opacification (VAO), VAO continues to be frequently observed in children; its incidence is higher in children than adults^(12)^. At younger ages, the higher number of mitotically active cells results in faster growth of the residual lens epithelial cells remaining after surgery^(6,13-15)^. In addition, the increased inflammation associated with surgical trauma at an immature angle, angle dysfunction or the possible disorganization of the anterior segment could explain the higher incidence of secondary glaucoma observed in infants^(1,16,17)^. According to a previous meta-analysis^(18)^, the timing of surgery, primary IOL implantation, and additional intraocular surgery appear to influence the risk of developing glaucoma^(9,19)^. Furthermore, the observation of long-term refractive errors in early infancy discourages the use of IOL implantation since the changes expected to occur in children’s corneal curvature and axial length are not considered in adult IOL power calculations^(20)^. While pediatric surgeons can estimate the acceptable long-term target refraction based on the patient’s age, the selection of an appropriate target remains challenging due the greater myopic shift.

Few studies have evaluated the optimal timing to perform primary IOL implantation in children^(21,22)^. The aim of this study was to analyze the safety of primary IOL implantation (performed using a single surgical technique) in children aged 5-24 months over a long-term follow-up period.

## METHODS

### Study population

This study was a retrospective review of children who underwent cataract surgery with primary in-the-bag IOL implantation between the ages of 5-24 months at the Sant Joan de Déu Hospital (Barcelona, Spain) from January 1, 2006 to January 31, 2016. This study followed the tenets of the Declaration of Helsinki and was approved by the Medical Institutional Review Board, with the oversight of the Sant Joan de Déu Hospital.

### Exclusion criteria

Patients with <1 year of follow-up after the procedure were excluded. Patients with incomplete datasets, children aged <5 months, and those with associated anomalies or previous ocular diseases (microcornea, microphthalmos, glaucoma, coloboma, uveitis) or systemic diseases were also excluded. IOL implantation is not routinely performed in our institution in children aged <5 months.

### Data collection

The collected data included the patient’s date of birth, sex, laterality, and age at the time of the cataract surgery, surgeon, the type and power of the implanted IOL, axial length prior to cataract surgery, adverse events, immediate refraction, corrected distance visual acuity (CDVA), and refraction at the final follow-up.

### Surgical technique

While under general anesthesia with dilated pupils, retina, corneal diameter, and IOL power of the patients were evaluated immediately prior to the cataract surgery. The IOL implantation was performed in patients without changes observed in a fundus examination and a horizontal corneal diameter ≥11 mm. The keratometry measurement was performed using a handheld keratometer (KM-500; Nidek Inc., Fremont, CA, USA), and the axial length was measured with contact biometry (Axis II A-scan; Quantel Medical) by the surgeon. The IOL power was calculated with the Sanders-Retzlaff-Kraff (SRK) II formula (before 2009) or Sanders-Retzlaff-Kraff Theoretic (SRK/T) (after 2009) with the aim of hyperopic correction (+6 diopter [D] for patients aged 5-12 months and +5 D for those aged 12-24 months) as the target refraction during the immediate postoperative period. All surgeries were performed by an experienced surgeon (MMB).

A superior clear cornea incision (3.2 mm) was generated, and an ophthalmic viscosurgical device was inserted into the anterior chamber. Manual anterior capsulorhexis was performed using Utrata forceps and followed by aspiration of the lens. Subsequently, a three-piece foldable acrylic IOL was implanted in-the-bag through the main incision with an injector. A stab incision was performed in the pars plana (2-3 mm from the limbus according to the patient’s age), and posterior central capsulotomy and anterior vitrectomy were performed with 20-gauge probe vitrectomy. The sclerotomy site was closed with a single buried 7-0 polyglactin (Vicryl) suture. After aspiration of the ophthalmic viscosurgical device, the incision was sutured with 10-0 nylon. At the end of the surgery, an antibiotic (cefuroxime 1mg/0.1ml) was injected into the anterior chamber, and an inferior subconjunctival injection of steroids (methylprednisolone) was administered.

### Postoperative assessment

Postoperatively, a topical combination of antibiotic and steroid drops was applied every 4 h for a week. The dose was subsequently tapered each week for another 4 weeks, and a cycloplegic eye drop was administered twice daily for 2 weeks. An oral steroid was administered for 7 days. Follow-up was performed 1 day, 1 week, and 30 days later, every 3 months for ≤1 year, and subsequently every 6 months following the surgery.

The visual acuity (VA) assessment depended on the child’s age. In children younger than 2 years, we used Teller Acuity Cards; in children aged 2-3.5 years, we used HOTV, a LEA Symbols test, or Allen’s Picture Cards. In children older than this age, HOTV, a Tumbling E test, or a Snellen chart was used. Refraction was performed during all ophthalmology appointments under cycloplegia with retinoscopy by the optometrist and confirmed by the ophthalmologist. Glasses or contact lenses were prescribed 15 days after surgery. During the follow-up, amblyopia and ocular alignment were treated as needed.

We diagnosed glaucoma based on the intraocular pressure (IOP >21 mmHg) and fundoscopy (cup-to-disc ratio changes). The IOP in collaborating patients was measured in the clinic. Children who did not collaborate had their IOP measured under sedation in the operating room. The Perkins tonometer was used. The presence of VAO was considered when lens material regrowth extended into the pupillary space and obscured the visual axis. Observation of any irregular pupil indicated the presence of corectopia. The main outcome measures included postoperative complications, refraction changes, and CDVA at the final follow-up.

### Statistical analysis

Quantitative variables are expressed as the mean and standard deviation. Qualitative variables are expressed as absolute and relative frequencies. A p-value <0.05 denoted statistically significant differences. Analysis of variance was used to compare the means of the spherical equivalent (SE) and VA according to the patient’s age at the time of the cataract surgery. The Kruskal-Wallis test was used to compare the follow-up time with the age at surgery. The chi-squared test was used to compare categorical variables. The generalized estimating equation method was used to compare the means of postoperative complications and SE at the final follow-up. A correlation analysis (Pearson’s r correlation) was performed between the follow-up time and the SE (D). A linear regression model was used to verify the possible relationships between VA and the following variables: complications SE, myopic shift, follow-up, and reoperation.

## RESULTS

Ninety-seven eyes were included in the study and 93 eyes in 68 patients were considered in the final analysis. Two selected children were excluded from the analysis since the IOL was placed in the ciliary sulcus due to posterior capsule rupture. One child had white cataracts in both eyes, while the other had a nuclear cataract. There were no additional surgeries required in these cases. The ages of these patients at the time of surgery were 7 and 9 months, respectively. The final VA was 0.4 logMAR in both eyes in one child, and 0.4 and 0.7 logMAR in the other.

The mean age of the children analyzed at the time of surgery was 15.06 ± 6.19 months (range: 5-24 months). Of the included children, 43 had unilateral cataracts (63.23%) and 38 were males (55.8%). In total, 47 of the cataracts were in the right eye (50.5%). The most frequent cataract morphology was nuclear in 34 eyes (36.6%), followed by total in 23 eyes (24.7%) and subcapsular 20 eyes (21.5%). Four eyes with persistent fetal vasculature were included, and the mean age at surgery was 8.0 ± 2.9 months (range: 5-12 months). There was no glaucoma reported in the patients with persistent fetal vasculature during the 4.1 years of follow-up.

### Visual and refractive outcomes

The SE at 30 days after the surgery averaged 3.62 ± 2.32 D (range: -1.50 to +9.75 D). The mean follow-up time was 70.85 ± 39.52 months (range: 12-173 months). At the final follow-up, the mean age of the children was 6.77 ± 3.31 years (range: 2-15 years), the mean SE was -0.09 ± 3.22 D (range: -8.75 to +7.00 D), and the mean CDVA was 0.48 ± 0.41 logMAR (range: 0-1.3 logMAR). The mean CDVA in the unilateral and bilateral cases was 0.64 ± 0.43 logMAR (range: 0.1-1.3 logMAR) and 0.33 ± 0.33 logMAR (range: 0.00-1.3 logMAR), respectively (p=0.000). In the bilateral cataract cases, we included both eyes as the eyes presented different complications, CDVAs, and refractive errors.

A generalized estimating equation was used to analyze the final SE and complications in the bilateral cases; differences were not statistically significant (p=0.371, p=0.108). The results according to the time of surgery (in months) are shown in table 1. There was a negative correlation between SE and follow-up.

**Table 1:** Outcomes according to age at surgery (in months)

Parameter	≤9 months (n=22)	10-18 months (n=37)	≥19 months (n=34)	p-value
Eyes, n (%)	22 (23.7)	37 (39.8)	34 (36.6)	-
Laterality, n (%) Unilateral	7 (31.8)	24 (64.9)	12 (35.3)	0.013^*^
Bilateral	15 (68.2)	13 (35.1)	22 (64.7)	
Mean follow-up (months) Median	54	72	61.5	0.085^**^
(IR)	(30-75.3)	(49.5-104)	(33-86)	
SE 1 month after surgery (D) Mean ± SD	5.58 ± 2.77	3.26 ± 2.03	2.74 ± 1.45	0.000^***^
(Range)	(0.50-9.75)	(-1.50-8.00)	(0.0-7.75)	
SE at the final follow-up (D) Mean ± SD	1.62 ± 4.05	-0.93 ± 2.77	-0.31 ± 2.68	0.000^***^
(Range)	(-8.00-7.00)	(-8.75-4.25)	(-7.00-5.00)	
CDVA (logMAR) Mean ± SD	0.44 ± 0.29	0.59 ± 0.47	0.37 ± 0.37	0.085^***^
(Range)	(0.1-1.1)	(0.00-1.3)	(0.00-1.3)	
Myopic shift (D) Mean ± SD	4.19 ± 2.86	3.64 ± 2.49	2.77 ± 2.00	0.092^***^
(Range)	(0.50-9.75)	(0.00-9.50)	(0.00-7.00)	
Postoperative complications, n (age in months) VAO	5 (7, 8, 8, 9, 9)	6 (11, 11, 12, 16, 17, 17)	2 (24, 24)	0.109^*^
Corectopia	1 (8)	3 (10, 14, 16)	1 (22)	
Pigments in IOL	0	2 (15, 16)	0	
Fibrin formation	0	1 (17)	0	
Strabismus (n, %)	9 (22.5%)	15 (37.5%)	16 (40%)	
Additional surgeries^****^ (n, %)	6 (33.3%)	9 (50%)	3 (16.6%)	0.323^*^

* Chi-squared test;

** Kruskal-Wallis test;

*** ANOVA;

**** excluded strabismus surgeries.

SE= spherical equivalent; SD= standard deviation; CDVA= corrected distance visual acuity; D= diopter; n= number; IR= interquartile range; VAO= visual axis opacification; IOL= intraocular lens.

**Figure 1:**
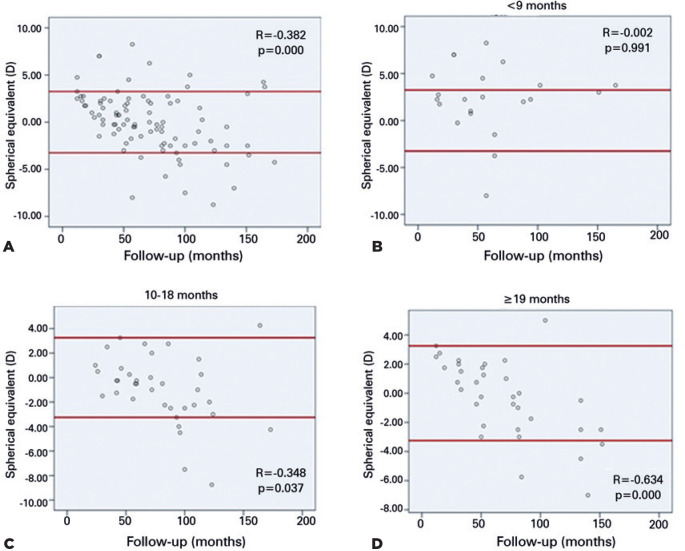
Correlation between the follow-up in months and spherical equivalent (SE) according to age at the time of surgery. (A) Total number of patients. (B) Group aged <9 months. (C) Group aged 10-18 months. (D) Group aged _≥_19 months. The scatter plots showing a negative correlation between the follow-up period and SE; longest follow-up presenting more myopic children.

To better analyze the refraction outcomes, the children were divided into groups according to their age at the time of surgery (Figure 1). The linear regression analysis revealed an association between VA and myopic shift (R^2^ =0.631), suggesting an average reduction in VA of -0.76 for each unit of increase in myopic shift. The highest myopic shift was observed in the infants who underwent surgery at ages 5 and 6 months. The final SE was >-3.50 (10.2%) in 11 eyes, ranged -3.50-0 (33.5%) in 36 eyes, ranged 0-+3.50 (33.5%) in 36 eyes, and was >+3.50 (9.3%) in 10 eyes.

### Adverse events and additional surgeries

The most frequent postoperative adverse event was VAO, which was observed in 13 eyes (13.97%), including eight eyes of children aged <1 year (61.53%) who underwent surgery. All children who developed VAO required enlargement of the posterior capsulotomy using a vitrectomy probe via the pars plana. The final CDVA was 0.41 ± 0.27 logMAR in these patients, and the second surgery was performed 19.92 ± 28.59 months (range: 1-108 months) after the first surgery. Corectopia was present in five eyes (5.37%) with a mean age at surgery of 14.0 ± 5.47 months (8-22 months), and all of these eyes required surgical repair 3.0 ± 3.9 months (range: 1-10 months) after the cataract surgery. The final CDVA was 0.62 ± 0.46 logMAR in these patients. Two patients exhibited IOL pigment deposits. Their final CDVA was 0.2 and 0.7 logMAR, respectively. One patient exhibited fibrin formation in front of the IOL that was resolved with administration of a topical steroid and cycloplegic. The final CDVA in this patient was 0.5 logMAR. At the final follow-up, strabismus was observed in 40 patients (43.0%). There was a positive correlation between the age at surgery and development of strabismus (r=0.015; p=0.886). Glaucoma or retinal detachment did not develop in any of the children.

The most frequently implanted IOL type was MA60BM (AcrySof; Alcon Laboratories, Inc., Fort Worth, TX, USA) in 73 patients (78.5%), followed by PC-60AD (Hoya, Tokyo, Japan) in 18 patients (19.3%) and Aaris EC-3 PAL (Aaren Scientific Adaptic Optics, France) in two patients (2.1%). There was no difference noted in complications according to the type of implanted IOL. The mean IOL power was 25.24±4.27 D (range: 12-30 D).

## DISCUSSION

Lens reproliferation into the visual axis was the most frequently observed complication in this study and mainly occurred in children who underwent surgery during the first year of life. This finding was in accordance with the literature, since the rate of VAO decreases with advancing age^(4-6,10)^. Corectopia and other adverse events had low incidences in this study^(10)^. The pars plana approach involves the avoidance of contact between the vitreous and trabecular meshwork, which could decrease inflammation and postoperative complications. Even performing posterior capsulotomy, VAO was the adverse event that required more additional surgeries. Moreover, most of the additional surgeries were performed during the first year of follow-up (72.2%).

In addition to greater inflammation, early cataract surgery increases the risk of glaucoma most likely due to disturbances in the angle and anterior segment, which are not yet well-formed in infants. Solebo et al. and Koch et al.^(6,23)^ found more cases of glaucoma in children who had undergone surgery prior to 6 months of life and that a young age at the time of surgery was the only factor associated with glaucoma. Trivedi et al.^(9)^ showed that the risk of glaucoma was higher in aphakic and pseudophakic eyes of pacients who underwent cataract surgery <4.5 months than older patients. In this study, safe results with IOL implantation in-the-bag were observed in children older than 5 months during the follow-up period (mean: 6.28 ± 3.37 years). This finding may be related to the completion of the formation of the angle in children aged 5-6 months. However, it is worth mentioning that the children included in this study could develop glaucoma in the future because this risk continues to exist.

Glaucoma can occur at any time following cataract surgery; thus, children with or without IOL implantation should be regularly examined for glaucoma throughout their lives^(24)^. The IATS in 5 years of follow-up, reported that the number of cases of glaucoma increased in both the aphakic and pseudophakic groups, with more new cases reported in the aphakic group. During the 1-year follow-up, there were more cases in the pseudophakic group; however, this result was not statistically significant. In addition, 22.2% of the glaucoma cases in the IOL group had an IOL placed in the ciliary sulcus^(10)^. Gawdat et al.^(25)^ reported that most cases of glaucoma occur during the first year of follow-up, and its incidence was higher in aphakic eyes than pseudophakic eyes (77% vs. 22%, respectively). Haargaard et al.^(26)^ showed that glaucoma cases continued to occur even >10 years after surgery. Those authors also found that glaucoma was related to the age at surgery in both aphakic and pseudophakic children (31.9% vs. 4.1%, respectively). Asrani et al.^(19)^ showed that the incidence of glaucoma was higher in aphakic than pseudophakic children; however, their follow-up period was longer in the aphakic group. Besides, children in the aphakic group were younger than children in the pseudophakic group, and the aphakic children were younger. It has been suggested to avoid vitreous contact with the anterior chamber during the placement of in-the-bag IOL, and an IOL provides mechanical support to the trabecular meshwork. However, this effect could be due to selection bias since aphakic children undergo surgery at an earlier age than pseudophakic children^(9,19,27)^.

Additionally, IOL implantation should be avoided in patients with microcornea and microphthalmos^(28)^. Some reports did not consider the preoperative corneal diameter a risk factor for the development of glaucoma^(9)^; however, the implantation of IOLs in corneas with small diameters can result in secondary glaucoma^(29)^. Measurement of the cornea diameter prior to all surgeries is routinely performed at our institution, and IOL implantation is performed only if the horizontal corneal diameter is ≥11 mm.

According to the linear regression analysis, clinicians should be aware that an increasing myopic shift may reduce VA. Spearman’s correlation analysis showed that a longer follow-up was associated with higher numbers of myopic cases, even though the myopic SE was low in most patients. In addition, there was a trend for a larger degree of myopic shift in children who underwent surgery earlier. However, based on refractive outcomes during the 6-year follow-up in this study, we report that, if the correct IOL power calculation is used, it is possible to achieve satisfactory SE and VA, even in young children.

By analyzing the 11 eyes with a final SE >-3.50, we observed that in all of these cases, the immediate postoperative refractive error was lower than expected based on age. Additionally, these cases exhibited more myopic shift (7.39 ± 1.45 D in 8.09 ± 3.44 years of follow-up; 14.0 ± 5.88 months at surgery). Among these patients, nine patients, all with unilateral cataract, were aged >9 months at the time of the cataract surgery (6.91 ± 1.13 D myopic shift in 9.0 ± 3.1 years of follow-up; initial SE: 2.41 ± 1.81; final SE: -5.55 ± 1.79). The other child with bilateral cataracts was 6 months old at the time of surgery and exhibited the greatest myopic shift recorded in this study (9.25 and 9.75 D in 5 years of follow-up; initial SE: 1.50 and 1.75; final SE: -8.00 and -8.25). The patient with the highest myopic SE (final SE: -8.75; 9.0 D myopic shift in 10 years of follow-up) among these patients also exhibited high myopia in the contralateral unoperated eye close to the myopia in the pseudophakic eye. Since more myopic shift is expected in patients aged <2 years, we recommend that calculations to target the hyperopic correction in the immediate refraction goal should be very carefully performed to better manage the refractive correction in the long-term. However, even with high myopia, these children obtained a satisfactory CDVA (0.48 ± 0.36 logMAR).

Although posterior capsulotomy and anterior vitrectomy were performed via the pars plana approach^(12)^ with in-the-bag IOL implantation in all patients, almost 14% of the children developed VAO in this study. We noted that, over time, a large capsulotomy is required to reduce VAO. Furthermore, when enlargement of the posterior capsulotomy is required, we suggest using a vitrectomy probe also via the pars plana.

A limitation of this study is its retrospective nature. Its strengths are that the surgeries were performed by one surgeon using a single technique and the use of a long follow-up period. The important characteristics of primary IOL implantation include the benefits it provides in the treatment of children with neurological delay and facial disorders, who are difficult to manage due to the necessity of use of heavy glasses or contact lenses. Our results may be extended to such cases.

The results of this study revealed that the rate of complications following primary in-the-bag IOL implantation was low in children aged 5-24 months. At this age glaucoma was not found, and most patients exhibited an acceptable refractive error during the follow-up; hence we support the use of primary three-piece IOL in-the-bag implantation. Careful calculation of the IOL power is essential in young children to obtain better long-term refraction outcomes.

### Highlights

This study has a long follow-up period (most children with >5 years of follow-up) and a large number of eyes, rendering it highly relevant to the field of pediatric cataract research.

The primary intraocular lens (IOL) was implanted in patients aged 5-24 months (an age group with outcomes that are scarcely considered by the currently available literature).

A safe period for initiating in-the-bag IOL implantation in infants was suggested.
